# Supplementary material for: MetaRibo-Seq measures translation in microbiomes
Source: Nat Commun. 2020 Jun 29;11:3268. doi: 10.1038/s41467-020-17081-z (PMC7324362; doi:10.1038/s41467-020-17081-z)
Supplement: Supplementary file 10 — Supplementary Data 7 [file 41467_2020_17081_MOESM10_ESM.zip › File2/Confidence_High_Taxonomy/306819_out.krona.html]

Javascript must be enabled to view this page.

members
magnitude
magnitudeUnassigned
count
unassigned
taxon
rank

306819\_out

10

superkingdom
2
10

phylum
976
10

class
200643
10

order
171549
10

815
1
family

816
1
genus


SRS098073\_contig\_number\_13987
species
469590
1

family
9
171552

9
838
genus


SRS024435\_contig\_number\_5721SRS049959\_contig\_number\_47293
2
1917878
species


SRS049446\_contig\_number\_5512SRS104912\_contig\_number\_4502
2
1947178
species


SRS013687\_contig\_number\_1840SRS023914\_contig\_number\_20776SRS144537\_contig\_number\_4508SRS149325\_contig\_number\_1379
4
159272
species

species
1
1262935

SRS013800\_contig\_number\_4482
